# A Novel Moderately Thermophilic Type Ib Methanotroph Isolated from an Alkaline Thermal Spring in the Ethiopian Rift Valley

**DOI:** 10.3390/microorganisms8020250

**Published:** 2020-02-13

**Authors:** Tajul Islam, Amare Gessesse, Antonio Garcia-Moyano, J. Colin Murrell, Lise Øvreås

**Affiliations:** 1 Department of Biological Sciences, University of Bergen, Thormøhlensgate 53 B, PO box, 7803, 5006 Bergen, Norway; tajisl@hotmail.no (T.I.); antonio.moyano@uib.no (A.G.-M.); 2 Bergen Katedralskole, Kong Oscars gate 36, 5017 Bergen, Norway; 3 Institute of Biotechnology, Addis Ababa University, Addis Ababa PO box 1176, Ethiopia; gessessea@biust.ac.bw; 4 Department of Biological Sciences and Biotechnology, Botswana International University of Science and Technology, Private bag 16, Palapye 10071, Botswana; 5 NORCE, Norwegian Research Centre, Marine Biotechnology, PO box 22, 5838 Bergen, Norway; 6 School of Environmental Sciences, University of East Anglia, Norwich Research Park, Norwich NR4 7TJ, UK; J.C.Murrell@uea.ac.uk

**Keywords:** Methanotrophs, Type Ib, moderate thermophile, alkaline thermal spring, pMMO, Ethiopian Rift Valley

## Abstract

Aerobic moderately thermophilic and thermophilic methane-oxidizing bacteria make a substantial contribution in the control of global warming through biological reduction of methane emissions and have a unique capability of utilizing methane as their sole carbon and energy source. Here, we report a novel moderately thermophilic *Methylococcus*-like Type Ib methanotroph recovered from an alkaline thermal spring (55.4 °C and pH 8.82) in the Ethiopian Rift Valley. The isolate, designated LS7-MC, most probably represents a novel species of a new genus in the family *Methylococcaceae* of the class *Gammaproteobacteria*. The 16S rRNA gene phylogeny indicated that strain LS7-MC is distantly related to the closest described relative, *Methylococcus capsulatus* (92.7% sequence identity). Growth was observed at temperatures of 30–60 °C (optimal, 51–55 °C), and the cells possessed Type I intracellular membrane (ICM). The comparison of the *pmoA* gene sequences showed that the strain was most closely related to *M.* *capsulatus* (87.8%). Soluble methane monooxygenase (sMMO) was not detected, signifying the biological oxidation process from methane to methanol by the particulate methane monooxygenase (pMMO). The other functional genes *mxaF*, *cbbL* and *nifH* were detected by PCR. To our knowledge, the new strain is the first isolated moderately thermophilic methanotroph from an alkaline thermal spring of the family *Methylococcaceae*. Furthermore, LS7-MC represents a previously unrecognized biological methane sink in thermal habitats, expanding our knowledge of its ecological role in methane cycling and aerobic methanotrophy.

## 1. Introduction

Methane plays a key role in the global carbon cycle, being 34 times more powerful as a greenhouse gas than CO_2_, and is the most substantial contributor to climate effect [1]. Abiogenic methane from underground reservoirs is produced in catalytic reactions at high pressure and temperature. Especially in geothermal habitats, a mixture of methane and other gases (known as natural gas) enter the Earth’s atmosphere as a part of volcanic gases and hydrothermal solutions through seeps, degassing of spring water and gas venting. Moreover, anaerobic microbials (the presence of methanogenic archaea) in hyperthermal hot springs also contribute formation and releasing of biogenic methane into the atmosphere [2,3,4]. In some parts of Ethiopia (the Great Rift Valley regions), natural methane is released through thermal springs nearby the Rift Valley lakes. Such lakes and thermally heated water sediments from hot springs may affect the community structure and diversity of microorganisms and may have a major influence in the global carbon cycle.

The study of aerobic methane-oxidizing bacteria (MOB) or methanotrophs is of special interest because of their significant ecological role in the global carbon cycle and natural reduction of methane emission to the atmosphere from many different ecosystems. Moreover, these microorganisms have the ability of utilizing methane as their sole energy and have a unique multicomponent enzyme system called methane monooxygenase (MMO), of which two distinct types, a particulate membrane-bound enzyme (pMMO) and a cytoplasmic soluble, membrane-free form (sMMO) have been described. These bacteria are found worldwide in nature and have been detected and isolated from a variety of thermal and non-thermal habitats [5,6,7,8]. Until now, taxonomical and molecular diversity studies of aerobic methanotrophs comprise the three phyla of *Proteobacteria, Verrucomicrobia* and *“Methylomirabilaeota”* (candidate phylum NC10). In the phylum *Proteobacteria*, methanotrophs are currently reclassified and defined into five distinct families: the gammaproteobacterial *Methylomonadaceae* (referred to as Type Ia), *Methylococcaceae* (Type Ib, formerly named as Type X), *Methylothermaceae* (Type Ic) and the alphaproteobacterial *Methylocystaceae* (Type IIa) and *Beijerinckiaceae* (Type IIb), based on their genomic comparisons (digital DNA-DNA hybridization (dDDH)), reconstruction of genome phylogeny, average nucleotide identity (ANI) and average amino acid identity [9,10,11,12,13]. Within the phylum *Verrucomicrobia* (sometimes referred also to Type III methanotrophs), only one family is defined as *Methylacidiphilaceae* that consists of, up to now, two genera: *Methylacidiphilum* and *Methylacidimicrobium* [14,15,16].

Although the majority of reported aerobic methanotrophs are mesophilic (optimal between 10 and 35 °C) and neutrophilic, their actual physiological tolerance ranges from 0 to 72 °C, pH from 1 to 11 and salinities up to 30%. In fact, several thermotolerant (growth up to 50 °C) and moderately thermophilic methanotrophs have also been described [9,13,17]. Our knowledge of truly thermophilic or moderately thermophilic proteobacterial methanotrophs (T_opt_ > 40 °C and T_max_ < 67 °C) is still limited compared to their thermotolerant (T_max_ < 50 °C), mesophilic (T_max_ < 42 °C) or psychrotolerant (T_max_ < 36 °C) counterparts. Only a few validly described species of thermophilic methanotrophs like *Methylothermus thermalis* (growth at 37−67 °C), *Methylothermus subterraneus* (growth at 37−65 °C) within the family *Methylothermaceae* and *Methylocaldum szegediense* (growth at 37−62 °C) within the family *Methylococcaceae*, could grow optimally above 55 °C [13,18,19]. The family *Methylococcaceae* presently comprises only seven phylogenetically associated genera: *Methylococcus, Methylocaldum, Methyloparacoccus, Methylogaea, Methylomagnum, Methyloterricola* and *Methylotetracoccus* [13,19,20,21,22,23,24,25]. In addition to these genera, the first isolated acid-tolerant moderately thermophilic (at a temperature range of 30−60 °C and at pH range 4.2−7.5) gammaproteobacterial methane oxidizers (methanotrophic isolates BFH1 and BFH2) were retrieved from a tropical topsoil with methane seeps habitat in Bangladesh, and possibly represent a novel new genus within the Type Ib methanotrophs. 16S rRNA gene phylogeny of both strains formed a cluster with the genus *Methylocaldum* as the closest described relative [26]. Furthermore, three novel isolates (strains GFS-K6, BRS-K6 and AK-K6) were also recovered from three different geographical regions and habitats: rice field soil, a methane seeps pond sediment from Bangladesh and a warm spring sediment from Armenia. But these microorganisms are mesophilic rather than thermotolerant and represent the members of Type Ib methanotrophs, and make, phylogenetically, a cluster together of the mesophilic genera *Methylomagnum* and *Methyloparacoccus* [27].

*Methylococcus capsulatus* strains, Texas and Bath, were the first reported thermotolerant methane oxidizers, growing up to 50 °C and at pH range 5.5−8.5, and were isolated from sewage sludge and geothermally heated water, respectively [21,28]. So far, strain *M. capsulatus* Bath is the most studied methanotroph and expanded knowledge of its ecophysiology, genetic and biochemistry. Bodrossy and colleagues, reported a bona fide novel thermophilic gammaproteobacterial methane-oxidizing bacterium (informally named as *“Methylothermus”* strain HB), which was recovered from underground hot springs in Hungary, and this bacterium represented the highest recorded growth temperature range at 40−72 °C, with an optimum at 62−65 °C, until now [29]. Sequence comparisons of both 16S rRNA and *pmoA* genes revealed that strain HB represents a novel genus of the Type Ic methanotrophs in the family *Methylothermaceae*, but unfortunately, this strain does not exist any longer [13]. Recently, the detection of a new gammaproteobacterial group of methanotrophs, distantly related to *Methylococcus* and *Methylocaldum*, in a Russian Far East thermal spring, provides new insights into the diversity and distribution of thermophilic methanotrophs [30]. Moreover, three thermoacidophilic strains, *M. infernorum, M. fumiolicum* and *M. kamchatkense*, were able to grow at temperatures of 37 to 65 °C and pH at up to 6.0. These were isolated from acidic geothermally heated soils and a hot spring, but they are members in the genus *Methylacidiphilum* of the phylum *Verrucomicrobia*. Verrucomicrobial methane oxidizers appear to be found only in acidic geothermal environments [14,15,16].

Several key functional molecular gene markers like *pmoA* (encoding a subunit of the particulate methane monooxygenase, pMMO: a copper-dependent enzyme), *mmoX* (encoding a subunit of the soluble methane monooxygenase, sMMO: an iron-dependent enzyme) and *mxaF* (encoding the large subunit of PQQ-dependent methanol dehydrogenase, MDH: a calcium-containing enzyme) were frequently applied for detecting and diversity analysis of C_1_-utilizing bacteria. Especially, the *pmoA* gene is often applied as a phylogenetic marker for identifying aerobic methanotrophs in various habitats [6,31]. Hitherto, no thermophilic methane oxidizers have been reported to exhibit both enzymes systems, indicating that methanotrophic cytoplasmic sMMO might not be existing in cells living above 55 °C [13,18,29]. The gene *cbbL*, encodes the large subunit of the ribulose-1,5-bisphosphate carboxylase/oxygenase (RuBisCo: an essential enzyme in the Calvin-Benson-Bossham cycle), which is responsible for autotrophic growth. This gene has been commonly utilized for analyzing marine and hypersaline microbial communities as well as studies of Type Ib methanotrophs [26,32,33,34].

Recovering of moderately thermophilic methane-oxidizing bacteria from alkaline thermal springs (pH 8.0 to 9.0) is still a challenging process. Little is known about the identity, community structure and distribution of these bacteria from such ecosystems.

In this study, we have isolated and characterized the first moderately thermophilic Type Ib methanotroph, recovered from an alkaline thermal spring sample from the Ethiopian Rift Valley. The new isolate likely represents a new genus within the family *Methylococcaceae* of the class *Gammaproteobacteria* and extends our knowledge of this group of microorganisms in these environments.

## 2. Materials and Methods

### 2.1. Sample Collection, Enrichments and Growth Conditions

**Figure 1:**
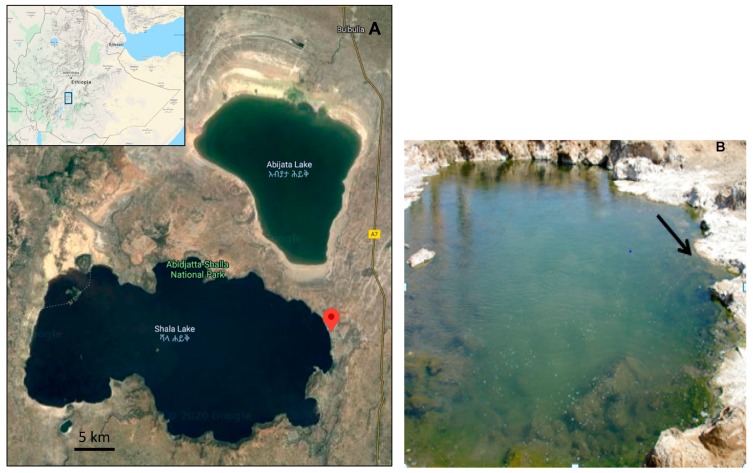
Satellite image of the Ethiopian Rift Valley region, showing the Lake Shalla and the location of the hot-spring. An insert on the map shows the location of the lake in Ethiopia (Google Maps, 2020) (**A**). A close-up photograph of the sampling site (**B**).

The alkaline-saline Lake Shalla is situated in the eastern Ethiopian Rift Valley [35], where extensive volcanic activity has disrupted drainage and thus helped create small shallow lake basins. Lake Shalla is a terminal lake with several thermal springs close by (Figure 1). The samples were collected in November 2007 from one of the largest ponds showing diffuse venting and emitting high temperature fluids. It is located 1561 m above sea level at a position 7°28′ 666′’N and 38°38′086′’E. Water and sediment samples were collected using a sterile pitcher connected to a stick at around 50 cm depth and around 50 cm from the shore of the pond. The samples were transferred to Falcon tubes that were filled completely with sediment and water from the thermal spring outlet.

For the enrichment and cultivation of moderately thermophilic methane oxidizers, 3 mL sediment slurry was added to 15 mL low-salt mineral medium (LMM) supplemented with KNO_3_ in 120 mL sterile serum flasks [27]. The pH of the LMM was adjusted to 7.0 with HCl or NaOH. In addition, a low-salt mineral medium, supplemented with either NH_4_ Cl (LMM-AC) or (NH_4_)_2_ SO_4_ (LMM-AS) adjusted to pH 7.0 was also utilized for methanotrophic enrichment cultures. The serum flasks were closed with butyl rubber stoppers with aluminum crimp seals and a mixture of 80% methane (purity 99.5%, Yara Praxair, Oslo, Norway) and 20% air aseptically was added through a syringe in the headspace. The same methane:air mixture was used in all growth experiments. The cultures were incubated at 55 °C for one week with shaking at 150 rpm in the dark.

### 2.2. Isolation and Purification of Strain LS7-MC

The primary enrichment culture was transferred up to five times and serial dilutions were made of the culture with LMM. 100 µL of dilutions (10^−5^ to 10^−8^) were spread onto gelrite solidified plates (20 g L^−1^; Gelzan^TM^ CM, Sigma-Aldrich) or agar (Difco) containing LMM. The plates were incubated for four weeks at 55 °C in jars filled with the methane and air mixture (4:1). No extra air was added in the jar. Colonies were picked and re-streaked onto fresh gelrite plates and re-incubated. Finally, one single colony was transferred to fresh liquid LMM with the methane:air mixture (4:1) in the headspace and incubated at 55 °C. These growth conditions with methane as the sole carbon source were routinely used for further cultivation of this isolate. The purity of the culture was evaluated by phase-contrast and electron microscopy, and repeated PCR amplification analysis of the partial 16S rRNA gene sequences. For further verification, the strain LS7-MC was also tested for lack of growth on acetate (10 mM), pyruvate (10 mM), succinate (10 mM), glucose (10 mM), ethanol (17 mM) and yeast extract (0.1%), streaking onto R2 A agar plates [36].

### 2.3. Naphthalene Assay, Acetylene Inhibition Test and Electron Microscopy

To test for the presence of sMMO, the naphthalene-oxidation assay was performed using a culture grown in liquid LMM without copper, as described by Graham and collaborators [37]. Acetylene has been applied as an inhibitor for completely blocking methane monooxygenase [38]. The effect of acetylene on strain LS7-MC was monitored by adding 4% (v/v) acetylene to the headspace after two days of incubation with methane. Three replicate flasks were used for the methane oxidation inhibition test and one flask without acetylene was used as a control. To validate the acetylene inhibition test, *M. kamchatkense* Kam1 and *M. capsulatus* Bath were used as positive controls under the same conditions [36,39]. The morphology of the strain LS7-MC was determined using phase-contrast microscopy (Nikon, Eclipse E400 microscope) and electron microscopy Jeol-1230. For electron microscopy, strain LS7-MC was grown at its optimum temperature (55 °C) and ultrathin sections were prepared as described previously [36].

### 2.4. Growth Conditions, Carbon and Nitrogen Sources

Growth of strain LS7-MC on various organic compounds was tested in liquid LMM supplemented with sterile substrates as potential carbon sources (acetate, pyruvate, succinate, glucose, lactate, maltose, ethanol and sorbitol) at a concentration of 10 mM. Growth on methanol, formaldehyde, methylamine, dimethylamine, formamide, glycerol, formate and yeast extract was examined using LMM containing the following variable concentration of the respective substrates: 0.01%, 0.05%, 0.1%, 0.25%, 0.5%, 0.75% and 1% (v/v). To determine the temperature range and the optimum temperature of strain LS7-MC, growth was tested at 16 different temperatures ranging from 20 to 70 °C. The effect of pH range was examined at the optimum temperature of 55 °C, with a range of 14 pH values from 5.0 to 10.0. The optical cell density was measured at 600 nm (Spectronic 21, Milton Roy Company). Nitrogen sources were tested by using liquid LMM, in which KNO_3_ (0.1 g L^−1^) was replaced by 0.1 g L^−1^ of NH_4_ Cl, (NH_4_)_2_ SO_4_, glycine, methylamine, dimethylamine and yeast extract. Strain LS7-MC was also tested in triplicate with nitrogen-free LMM, where N_2_ from the air (20% air in the headspace) was the only nitrogen source. Exponential-phase cells (1:10 dilution) were supplemented into LMM (without KNO_3_) and this culture was transferred three times for excluding any trace of nitrate from the original inoculum. Growth was examined after two weeks of incubation at 55 °C. The generation time and the specific growth rate were calculated from exponential phase of growth under optimal growth conditions. For testing heat-resistance, a 4-week-old culture of strain LS7-MC was heated at 80 °C for 10 min. Growth of strain LS7-MC was also tested without vitamins in LMM. The strain was examined for growth on LMM (KNO_3_) without added vitamins and CuSO_4_ · 5 H_2_ O (0.2 g L^−1^). Exponential-phase cells (1:10 dilution) were added to the medium and continued to grow until three passages. NaCl tolerance and the test of antibiotic sensitivity of strain LS7-MC were followed as delineated previously [26]. Growth was performed with methane for one week at 55 °C.

### 2.5. Fatty Acid Analysis

For fatty acid analysis, cultures of strain LS7-MC grown at optimum temperature (55 °C) and with cell densities 10^8^ cells/mL were delivered to DSMZ (Deutsche Sammlung von Mikrooganismen und Zellkulturen GmbH, Germany), where the samples were processed by harvesting, saponification, methylation, extraction and base wash prior to gas chromatography analysis. The fatty acid database of the Microbial Identification System (MIS) was employed for comparing with the fatty acid patterns of strain LS7-MC.

### 2.6. PCR Amplification and Southern Blot Hybridization of Functional Genes

Genomic DNA was extracted using GenElute Bacterial Genomic DNA kit (Sigma). The 16S rRNA genes were amplified with the universal bacterial primers 27 f and 1492 r, using a Veriti 96-well Thermal Cycler (Applied Biosystems). The PCR was performed using Dynazyme^TM^ High-fidelity DNA polymerase (Finnzymes) and the PCR and sequencing protocols were followed as previously described [27]. The functional genes *pmoA, mmoX, mxaF, cbbL* and *nifH* (a gene responsible for nitrogen fixation) were amplified using the same PCR protocol as described above for the 16S rRNA gene. Primers used in this study are listed in Supplementary Table S1. Furthermore, amplified fragments of 16S rRNA genes, *pmoA* and *cbbL* genes were cloned using a TOPO-TA Cloning Kit (Invitrogen). The clones were screened for correct inserts and the fragments were sequenced. For confirmation of pMMO and sMMO, the Southern blotting technique was applied. Genomic DNA from strain LS7-MC, *M. kamchatkense* Kam1 (as a negative control) and *M. capsulatus* Bath and *Methylococcaceae* strain BFH1 (as positive controls) was extracted. Then, DNA was digested with EcoRI and HindIII. Hybridization probes and the further process were followed as previously described [32,36].

### 2.7. Phylogenetic Analysis and Nucleotide Sequence Accession Numbers

16S rRNA gene sequences, and PmoA, MxaF and CbbL protein sequences of strain LS7-MC were compared with available sequences in the GenBank database using the NCBI tools (Blastn and Blastp). To perform phylogenetic analysis, 16S rRNA gene sequences and deduced amino acid sequences of PmoA were aligned using the CLUSTAL W algorithm. Distances were computed and phylogenetic trees were constructed using the following methods: Neighbor Joining (NJ), Maximum Likelihood (ML), Minimum-Evolution (ME), and the following models: Maximum Composite Likelihood, Kimura 2-parameter, Tamura 3-parameter, Jukes–Cantor, Jones Taylor-Thornton (JTT), Poisson and Dayhoff, which are implemented in the MEGA7 software package. The confidence of the trees was determined by 1000 bootstrap replications [40]. The phylogenetic analysis of 16S rRNA and PmoA involved 1424 nucleotides and 167 amino acid sequences, respectively. The nearly complete 16S rRNA gene sequences and the partial sequences of the genes *pmoA*, *mxaF*, *cbbL* and *nifH* of the strain LS7-MC have been deposited in GenBank under the accession numbers KP771709, KP828775, KP843192, KP843193 and KP843194, respectively.

## 3. Results

### 3.1. Isolation of a Moderately Thermophilic Methylococcus-Like Methanotroph

The enrichments of moderately thermophilic methane oxidizers were achieved from an alkaline hydrothermal spring that has been present for the last 80 years. The in-situ temperature, the pH and the conductivity of the thermal spring were 55.4 °C, 8.82 and 8.6 S/m, respectively. Three separate enrichments, LMM (KNO_3_), LMM-AC (NH_4_ Cl) and LMM-AS ((NH_4_)_2_ SO_4_) were set up. After two weeks of incubation at 55 °C, the enrichment with LMM showed that cells’ turbidity and microbial growth was confirmed by phase-contrast microscopy. Growth on LMM-AC (NH_4_ Cl) and LMM-AS ((NH_4_)_2_ SO_4_) were not evident even after three weeks of incubation in the same culture conditions. Diluted LMM enrichment cultures were spread on gelrite plates and incubated for 10 days. Two different types of colonies were observed. One type was small white colonies about 0.6–0.8 mm in diameter and the other type was shiny and slightly larger, at about 1.2–1.5 mm in diameter. No such colonies appeared on agar plates. The small white colonies stopped growing after 10 days, but the cells were viable for about four weeks of incubation with methane. Using phase-contrast microscopy, the small white colonies were comprised of coccoid cells, whereas the larger and shiny colonies showed small rod-shaped cells. Physiological and taxonomic depiction of the ‘rod-shaped’ strain is currently under investigation (Islam et al., in prep.). For this current study we selected the small white colonies with coccoid cells for further characterization. The final isolate, designated LS7-MC, grew on methane or methanol as the sole carbon and energy source. It did not grow on ethanol, acetate, pyruvate, malate, succinate, methylamine, glucose, fructose or yeast extract, indicating that the isolate is an obligate aerobic methanotroph. No heterotrophic contaminants grew on these media, thus verifying the purity of the strain. Furthermore, repeated PCR amplification analysis of the partial 16S rRNA gene sequence from the methane and methanol grown cultures yielded the same sequence, confirming the purity of LS7-MC.

### 3.2. Growth and Physiological Characteristics of LS7-MC

The temperature range for growth was between 30 and 60 °C, and no growth was observed at 25 or 62 °C. The optimum growth temperature was 51−55 °C at pH 7.0. The cells did not appear to be heat-resistant. Growth occurred between pH 6.0–9.3 but not at pH 5 or 9.5. The optimal pH was between 7.0 and 7.5. Growth was not obtained under aerobic conditions in the absence of methane or under anaerobic conditions in the presence of methane. Furthermore, the isolate grew only in the presence of very low methanol concentrations, between 0.05% and 0.25%. Strain LS7-MC did not grow on LMM-AC (NH_4_ Cl), LMM-AS ((NH_4_)_2_ SO_4_) and nitrogen-free compounds, except LMM (KNO_3_). Only nitrate in the medium was used as a nitrogen source. This indicated that the addition of nitrate has strong effects on methane consumption for growth or that the strain was possibly sensitive to ammonium salts. Multicarbon compounds completely prevented growth on methane or methanol at pH 7.0. The strain was able to grow to an OD_600_ value of 1.2 on methane. The generation time and the specific growth rate of a culture grown on methane were estimated to 6 h and 0.115 h^−1^, respectively. No growth was observed after 10 days of incubation for each of these passages in LMM medium without added CuSO_4_ or vitamin, indicating that both vitamins and Cu^+2^ are essential for growth. NaCl was not required for growth, but the isolate could grow on medium supplemented with 0.1%–0.25% NaCl (w/v). No growth occurred at concentrations above 0.5% NaCl (w/v). All tested antibiotics (ampicillin 10 µg mL^−1^, tetracycline 10 μg mL^−1^, kanamycin 30 µg mL^−1^, streptomycin 10 µg mL^−1^, erythromycin 10 µg mL^−1^ and nalidixic acid 30 µg mL^−1^) suppressed growth of LS7-MC. The addition of acetylene (4%) to the headspace of exponentially growing cultures, resulted in inhibition of methane oxidation, and further growth of strain LS7-MC stopped. This demonstrated the presence of the functionally active methane oxidizing enzyme typical for MOB. Major characteristics of the strain LS7-MC with other described obligate Type Ib methanotrophic genera or species of the family *Methylococcaceae* are presented in Table 1.

### 3.3. Microscopic Observations

**Figure 2:**
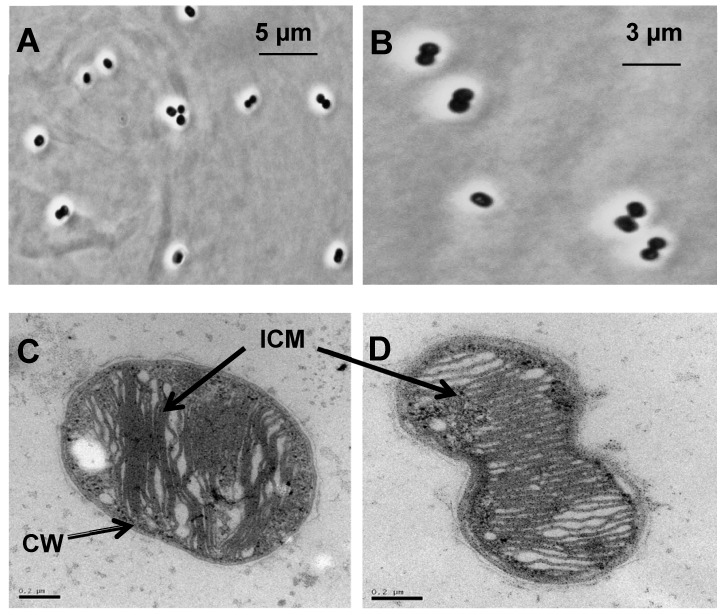
Morphology of the methanotrophic strain LS7-MC. (**A** and **B**) Phase-contrast photomicrograph of cells grown in low-salt mineral medium (LMM) medium with methane at 55 °C for 5 days. Transmission electron micrograph (TEM) of the strain LS7-MC shows (**C**) a single cell and (**D**) a diplococcus. Ultrathin sections showing extensive intracytoplasmic membrane (ICM) systems and a typical Gram-negative cell wall (CW). Bars, 0.2 µm.

Coccoid-type cells were observed by phase contrast microscopy (Figure 2A,B). The cells occurred individually or in pair (diplococcus) with a diameter and a length of 0.9–1.2 µm (Figure 2C,D). During cell division, an ellipsoid form appeared which turned into a diplococcus. This morphology was also seen in a thermophilic methanotrophic strain HB [29]. The strain was non-motile and multiplied by binary fission. Flagella were not apparent by transmission electron microscopy (TEM). Analysis of ultrathin sections by TEM revealed a typical Gram-negative cell wall structure and moreover, the presence of extensive intracytoplasmic membrane (ICM) (Figure 2C,D). Cells became elongated when the growth temperature was above 55 °C.

### 3.4. Phospholipids Fatty Acids (PLFA) Composition

The major fatty acids of strain LS7-MC are shown in Table 2 together with those of related Type Ib methanotrophs of the family *Methylococcaceae*. The fatty acid composition of this novel organism was revealed as comparable to other strains of the species *M. capsulatus, Methylocaldum* spp., *Methylotetracoccus oryzae*, *Methylogaea oryzae, Methyloparacoccus murrellii, Methylomagnum ishiwaze* and *Metyloterricola oryzae* regarding the two predominant fatty acids (C16:0 and C16:1ω7 c). In strain LS7-MC C16:0 (47.75%) and C16:1ω7 c (40.95%) accounted for 88.7% of the total amount of fatty acids. C16:0 is a major fatty acid in thermophilic, thermotolerant and mesophilic methanotrophs of the Type Ib genera like *Methylocaldum*, *Methylococcace* strain BFH1, *Methylococcus, Methyloterricola* and *Methylogaea* (30−60%), whereas mesophilic methanotroph genera (e.g., *Methylotetracoccus*, *Methyloparacoccus* and *Methylomagnum*) contain less than 24% of C16:0 [22]. Type Ia mesophilic or psychrotolerant genera in the family *Methylomonadaceae* (*Methylosoma*, *Methylobacter* and *Methyloglobulus*) exhibit more than 55% of C16:0 [43]. Hitherto, such high amounts of C16:1ω7 c found in strain LS7-MC have not been reported in any other described thermophilic or thermotolerant Type Ib methanotrophs. *Methyloparacoccus*, *Methylomagnum* and *Methyloterricola* as mesophiles, contain 54%, 47% and 27% of C16:1ω7 c, respectively. The following fatty acids were detected in less amounts (1% to 6%): C17:0 *cyc*, C16:0 3-OH and C18:1*ω*7 *c*, accounting for 9.0% of total fatty acids. In general, fatty acid profiles from other related methanotrophs differ significantly from that of strain LS7-MC.

### 3.5. Detection of Functional Genes

All amplification reactions of functional genes gave positive results except for the gene *mmoX*. Strain LS7-MC did not show any positive results in the naphthalene assay, verifying the absence of sMMO. Southern blotting analysis of genomic DNA also showed no positive signals with the *mmoX* probe, whereas the *pmoA* probe yielded positive signals (Supplementary Table S2). These results confirmed that strain LS7-MC does not contain the soluble form of MMO.

### 3.6. Phylogenetic Analysis of 16S rRNA and Functional Genes

**Figure 3:**
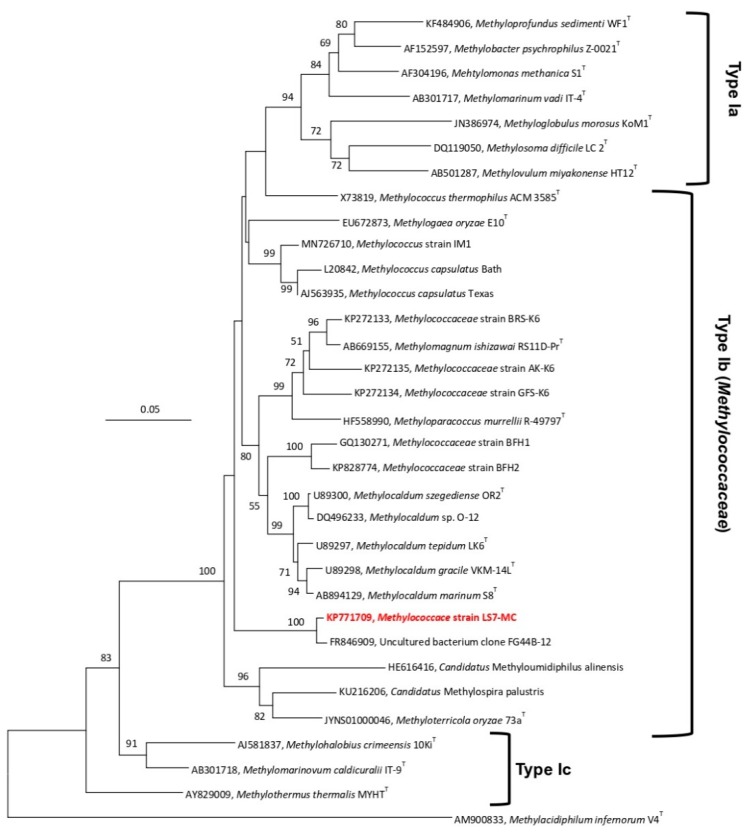
16S rRNA gene-based phylogenetic tree, using the Neighbor-Joining method, showing the phylogenetic position of strain LS7-MC (highlighted in bold red) within the family *Methylococcaceae* (Type Ib) of the class *Gammaproteobacteria*. The evolutionary distances were computed using the Maximum Composite Likelihood method and are in the units of the number of base substitutions per site. *Methylacidiphilum infernorum* V4^T^ (AM900833), a thermoacidophilic verrucomicrobial methanotroph, was used as an outgroup. Evolutionary analyses were conducted in MEGA7 [40]. The scale bar represents 0.05 changes per nucleotide position. Less than 50% of bootstrap values (1000 replicates) are not shown.

A nearly complete sequence of the 16S rRNA gene (1424 bp) was obtained. Using Blastn search of the 16S rRNA gene sequence, strain LS7-MC showed a high sequence similarity (98.7%) to an uncultured bacterium clone (FG44 B-12) from a subterranean radioactive thermal spring in the Austrian central Alps (‘‘Franz-Josef-Quelle’’ in Bad Gastein; GenBank Accession No. FR846909) [44]. The strain showed 92.5% sequence similarity to uncultured bacterial clones from fracture water of a gold mine borehole in the USA (JX434172, JX434181, JX434257-58, JX434183, JX434188-89, JX434195, JX434201, JX434207, JX434214, JX434216, JX434220-22, JX434225), and a soil sample from Bugok geothermal in South Korea (MN726710). Low sequence similarity values were also found at 93.1% similarity to uncultured bacteria clones from industrial sugarcane bagasse feedstock piles [45], and *Methylococcus* sp. strain IM1 from geothermal field soils. The closest extant strains were *M. capsulatus* strain Bath (92.7% similarity), acid-tolerant moderately thermophilic strains BFH1 and BFH2, *M. szegediense* strain OR2 (92.9%), *Methylocaldum* sp. O-12 (92.8%) and *Methylocaldum* sp. E10 (92.9%). Analysis of pairwise alignment 16S rRNA gene sequence of the strain LS7-MC and the closest extant strains showed maximum sequence similarity (92.7%) with *M. capsulatus* strain Bath, which is a thermotolerant methanotroph of the family *Methylococcaceae*. Further analysis exhibited 91.2–92.4% sequence similarity to other strains (Supplementary Table S3). These results suggested that strain LS7-MC may represent a new member of the Type Ib methanotrophs rather than Type Ia (*Methylomonadaceae*) or Type Ic (*Methylothermaceae*) in the class *Gammaproteobacteria*. In the Neighbor-Joining tree of 16S rRNA gene (Figure 3), strain LS7-MC formed a phylogenetically separate linage within the closest genera of *Methylococcus*, *Methylocaldum*, *Methylogaea* and *Methyloparacoccus, Methylomagnum, Methylotetracoccus* and *Methyloterricola.* This topology was also verified with the Maximum-Likelihood (Supplementary Figure S1) and Minimum-Evolution (Supplementary Figure S2) trees, suggesting that strain LS7-MC is not a species within any of the known genera of the Type Ia, Type Ib or Type Ic methanotrophs.

**Figure 4:**
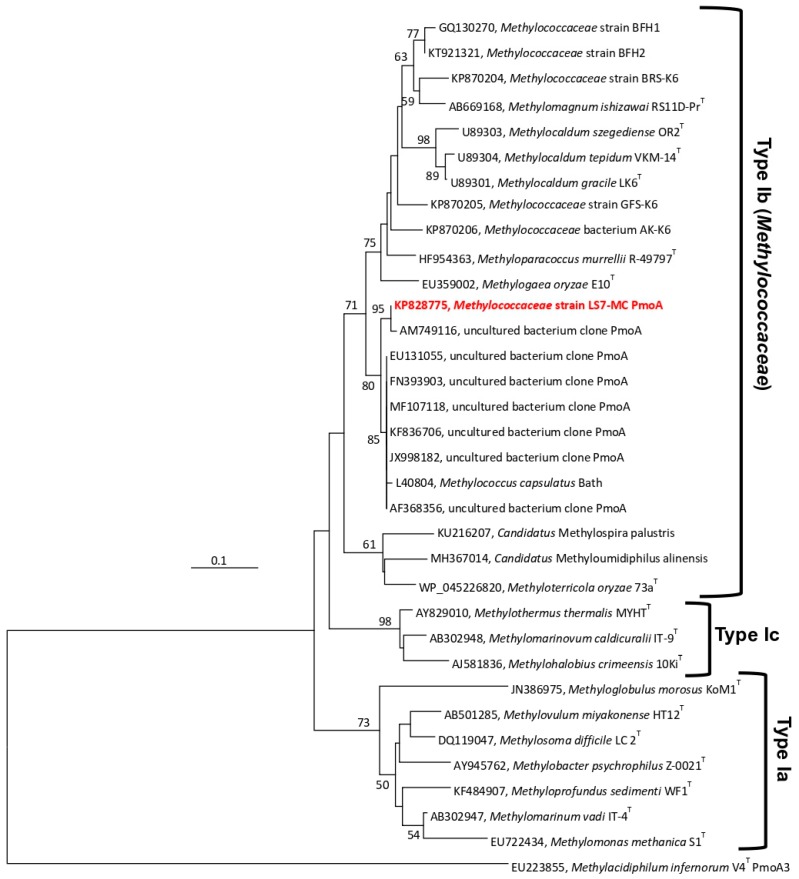
Phylogenetic dendrogram based on derived deduced PmoA amino acid sequences showing the position of strain LS7-MC (highlighted in bold red) and other described gammaproteobacterial methanotrophs. The evolutionary distances were computed using the Poisson correction method and are in the units of the number of amino acid substitutions per site. *Methylacidiphilum infernorum* V4 ^T^ PmoA3 (EU223855), a thermoacidophilic verrucomicrobial methanotroph, was used as an outgroup. Evolutionary analyses were performed in MEGA7 [40]. The scale bar represents 0.1 changes per nucleotide position.

Based on the *pmoA* gene analysis and Blastn search, strain LS7-MC showed 93.8% (99.4% amino acid level) sequence identity to the partial *pmoA* gene sequences of a DGGE band of an uncultured bacterium clone in an Austrian radioactive thermal spring (AM749116) [46]. Lower sequence identities (88.2–91.2%) were found compared to many uncultured bacteria from different ecosystems, such as wetland soil (JQ038175), hot water (AF533666), diverse soils (MF107118), subsurface borehole water (KF901437), hot spring (KF836706), landfill cover soil (JX998182), forest soils (FN393903), coal mine soil (EU131055) and Danish soils (AF368356). The *pmoA* gene of the strain LS7-MC, based on pairwise sequence analysis, revealed 87.8% identity (99.4% at amino acid level) to *M. capsulatus* Bath, 79.7–82.7% identity (92.3–94.7% at amino acid level) to *Methylocaldum* spp. (*M. szegediense* OR2 ^T^, *M. tepidum* LK6 ^T^, *M. gracile* VKM 14 L^T^, and *M. marinum* S8 ^T^), 84.6% identity (96.2%) to *M. murrellii* R-49797 ^T^ and 79.0% identity (95.3%) to *M. oryzae* E10 ^T^ (Supplementary Table S3). The phylogenetic analysis of partial derived PmoA amino acid sequences showed that the strain LS7-MC was clustered along with *M. capsulatus*, *Methylocaldum* spp., *M. murrellii*, *M. oryzea* and *Methylococcaceae* strains GFS-K6 and AK-K6 (Figure 4). The same topology was also found using Maximum-Likelihood (Supplementary Figure S3) and Minimum-Evolution (Supplementary Figure S4) trees, suggesting that the PmoA-based and 16S rRNA-based phylogenies generated a consistent position between strain LS7-MC and other cultivated Type Ib methanotrophs of the family *Methylococcaceae*. Analyses of the *mxaF* gene of the strain LS7-MC exhibited lower homology (83.6–85.9%) to several uncultured bacteria from coal mine soils (KF031213-15, KF031234, EU131063 and EU131061). The closest related genera, based on the *mxaF* gene (555 bp) and MxaF protein sequences (185 amino acids) of strain LS7-MC, were *Methylococcus*, *Methylocaldum*, *Methyloparacoccus* and *Methylogaea*, demonstrating 81.5–85.7% identity at the DNA sequences level and 96.5–97.8% similarity at the amino acid level (Supplementary Table S4). Pairwise partial-derived CbbL protein sequences (101 amino acids) comparison showed 98.1% identity with CbbL from *M. capsulatus* strain Bath (AF447860) and 95.0% with CbbL from *M. szegediense* (WP_026609010).

## 4. Discussion

Research on methane-oxidizing bacteria has mainly focused on low-temperature ecosystems. Most of the more than 50 validates species are either mesophilic or psycrophilic. Only a few thermophilic or moderately thermophilic species (8 species of proteobacterial and 3 species of verrucomicrobial methanotrophs) with optimal growth temperature between 50 and 55 °C have so far been reported [13,14,26]. In this study, enrichments for aerobic methanotrophs were established by inoculating sediment slurry from a thermal spring close to an alkaline lake (Lake Shalla) in the Ethiopian Rift Valley. From these enrichments, a novel moderately thermophilic Type Ib methanotroph, termed LS7-MC, was retrieved. This novel isolate shows an obligate aerobic methylotrophic growth with methane and methanol as the sole carbon and energy sources. According to 16S rRNA, PmoA, MxaF, CbbL and NifH sequence analyses, strain LS7-MC is most closely related to the Type Ib thermotolerant and moderately thermophilic methanotrophic bacteria within the family *Methylococcaceae*. Both the particulate methane monooxygenase gene *pmoA* and the methanol dehydrogenase gene *mxaF,* routinely used as functional and phylogenetic biomarkers for methanotrophic proteobacteria in natural environments [31,47], were detected in strain LS7-MC, indicating that methanotrophic proteobacteria are more widespread in thermal environments than previously thought. Furthermore, the difference in 16S rRNA gene sequences between strain LS7-MC and other related validated Type Ib methanotrophic genera ranges between 7% and 10%. The lack of soluble methane monooxygenase gene mmoX implies another significant difference between the thermotolerant genus *Methylococcus* and the strain LS7-MC presented here. These results ascertain that strain LS7-MC is, most probably, not a new species or subspecies of the genus *Methylococcus* or other genera of the families Candidate *Methylomonadaceae*, *Methylococcaceae* or *Methylothermaceae*. The 16S rRNA gene analyses suggested that this strain most probably represents a new genus in the methane-oxidizing bacterial family *Methylococcaceae* of the class Gammaproteobacteria.

Thermotolerant and moderately thermophilic proteobacterial methanotrophs have been found in various ecosystems from thermal spring sediments, subsurface hot aquifer, tropical landfill wetlands, methane seeps topsoil, compost and marine sediments [17,18,26,42]. The existence of these bacteria was supported by phylogenetic analyses of genes (like 16S rRNA, *pmoA, mxaF* and *mmoX*), through cultivation efforts and bacterial community analyses (using DNA-SIP, metagenomics, metatranscriptomics and next-generation sequencing) [48,49,50,51]. The comparative analysis of the 16S rRNA gene showed a relatively high sequence identity (>98%) to a clone of subsurface water sample (average pH 7.88) from an Austrian radioactive thermal spring [44]. The high sequence similarity of this clone to strain LS7-MC may indicate common physiology and metabolism properties between the strain LS7-MC and the uncultured thermal spring bacterium. Moderately thermophilic methanotrophs related to strain LS7-MC could be present in the central Austrian radioactive thermal spring and possibly in other related habitations of radioactive geothermal, and *pmoA* gene sequences obtained from the same thermal spring showed 93.8% sequence identity to *pmoA* of the strain LS7-MC. This is an indication that moderately thermophilic gammaproteobacterial methanotrophs may play a significant role in the carbon cycle in such subsurface thermal spring ecosystems. A relatively lower percentage identity of 16S rRNA, *pmoA* and *mxaF* sequences have also been detected in coal mine, diverse soils, wetland and landfill cover soils as well as hot spring water [19,52,53,54], suggesting that *Methylococcus*-like mesophilic or moderately thermophilic methanotrophs might be found in these various environments.

Strain LS7-MC required vitamins and copper for consistent growth and was negative with the naphthalene-oxidation assay. The lack of sMMO was also verified using the Southern blotting technique and PCR amplification. These central observations have also been described in other thermophilic proteobacterial isolates such as ‘*Methylothermus’* strain HB [29], *Methylothermus* spp. [18,48] and thermophilic *Methylocaldum* spp. [17], as well as the verrucomicrobial *M. kamchatkense* Kam1 [36], which suggests that genes encoding soluble methane monooxygenase most probably do not exist in moderately thermophilic or thermophilic proteobacterial methanotrophs or verrucomicrobial thermoacidophilic methanotrophs [14]. On the other hand, within Type Ib methane-oxidizers, thermotolerants such as *M. capsulatus* and *M. marinum,* and mesophilic *M. ishizawai* (growth range 4–37 °C) possess both sMMO and pMMO enzyme systems [13,42,55].

## 5. Conclusions

We have retrieved an obligate moderately thermophilic Type Ib methanotroph that belongs to the family *Methylococcaceae* of the class *Gammaproteobacteria*. This new isolate is a *Methylococcus*-like bacterium that contains the particulate methane monooxygenase (pMMO) but does not contain soluble methane monooxygenase (sMMO) in the methane oxidation process. Based on the physiological, biochemical and genotypic properties, strain LS7-MC most probably represents a novel genus within the family *Methylococcaceae*. This strain also denotes a previously unrecognized biological methane sink, diversity of methane oxidation and on the adaptation of this process to alkaline thermal habitats. Furthermore, this finding will increase our knowledge of methanotroph ecology and its involvement to global cycles of carbon and nitrogen, and the thermophilic nature of this strain possibly makes a considerable candidate for potential biotechnological applications [56]. Additional studies regarding the molecular biology, biochemistry and whole genome of LS7-MC are needed to provide insight into how biological methane oxidation processes and mechanisms are regulated in alkaline thermal ecosystems.

## Figures and Tables

**Table 1:** Comparison of the major characteristics of the strain LS7-MC with other described Type Ib methanotrophs of the family *Methylococcaceae*. Strains: **1**, This study; **2**, *Methylococcus capsulatus* strain Bath [20,41]; **3**, *Methylocaldum* spp. [17,19]; **4**, *Methylococcaceae* strain BFH1 [26]; **5**, *Methylotetracoccus oryzae* strain C50 C1 ^T^ [25]; **6**, *Methylogaea oryzae* E10 ^T^ [21]; **7**, *Methyloparacoccus murrellii* R-49797 ^T^ [22]; 8, *Methylomagnum ishizawai* RS11 D-Pr^T^ [23]; **9**, *Methyloterricola oryzae* 73 a^T^ [24]; +, positive results; –, negative results; nd, not determined.

Characteristic	1	2	3	4	5	6	7	8	9
Cell morphology	Coccoids	Coccoids	Rods-pleomorphic	Coccoids	Coccoids	Curved rods	Coccoids	Rods	Coccus
Temperature condition	Moderately	Thermotolerant	Thermophilic/	Moderately	mesophilic	Mesophilic	Mesophilic	Mesophilic	Mesophilic
	thermophilic		thermotolerant	thermophilic					
Internal membranes	Type I	Type I	Type I	Type I	Type I	Type I	Type I	Type I	Type I
Motility	−	−	+/−	−	−	+	−	+	+
Pigmentation	White	Yellow	Brown/cream	White	white	White	White	White	White
pMMO	+	+	+	+	+	+	+	+	+
sMMO	−	+	−/+^a^	−	−	−	−	+	−
*mxa*F	+	+	+	+	+	+	+	+	+
*cbbL* (RuBisCo)	+	+	+	+	+	nd	nd	+	+
*nifH* gene/N_2_-fixation	+	+	+	+	+	+	−	−	+
Growth on N-free medium	−	+	nd	−	nd	−	−	−	+
**Range of temp. (optimal)**	**30−60** **(51−55)**	**20−47** **(42−45)**	**20−61** **(42−55)**	**30−60** **(51−55)**	**4−30** **(18−25)**	**20–37** **(30–35)**	**20–37** **(25–33)**	**20–37** **(31–33)**	**15–45** **(27–37)**
^o^C	6.0−9.3	5.5−7.5	6.0−8.5	4.2−7.5	6−8	5–8	5.8–9.0	5.5–9.0	4.6–7.5
pH growth range (optimal)	(7.0−7.5)	(6.5)	(7.0−7.2)	(5.5−6.0)		(6.5– 6.8)	(6.3–6.8)	(6.8–7.4)	(7.0–7.5)
Growth on methanol (0.1%)	+	+	−	+	+	+	–	–	–
Vitamin required	+	−	−	−	−	–	–	+/–	–
Growth with C1									
compounds^b^	−	+	−	−	+	nd	nd	nd	nd
G+C content (mol%)	nd	62.5	57−59	62.7	62.77	63.1	65.6	64.1	61.0
G+C content (mol%)^c^	59.4	60.3	56.7^d^	59.5	nd	57.5	57.8	61.4	55.1^d^
**Isolation source (pH)**	**Alkaline ** **thermal spring (pH 8.82)**	**Hot spring** **(pH 6.0)**	**Manure, silage** **(pH 6.0)**	**Tropical topsoil** **(pH 5.0)**	**Rice field** **(pH 6.0)**	**Rice field** **(pH 5.8)**	**Pond water** **pH (7.1–8.4)**	**Rice field** **pH (6.3)**	**Rice plants** **pH (6.0–8.4)**

^a^ Only *Methylocaldum marinum* strain S8 ^T^ possessed the soluble methane monooxygenase gene [42]. ^b^ Formamide, formate and methylamine (0.1% w/v). ^c^ 16S rRNA, *pmoA*, *mxaF*, *cbbL* and *nifH* genes sequences were used for computing measurement of DNA G+C content (mol%). ^d^ Only 16S rRNA and *pmoA* sequences of *Methylocaldum szegediense* OR2 ^T^ and *Methyloterricola oryzae* 73 a^T^ were applied. Bold values show significant range of temperatures and pH of isolation sources among Type Ib methane oxidizers.

**Table 2:** Phospholipid fatty acids comparison of the strain LS7-MC and other related gammaproteobacterial.

Fatty acids	1	2	3	4	5	6	7	8	9
C12:0						2.11			
C13:1	0.50							0.27	
iC14:0				0.59					
C14:0	0.79	0.8−0.62	1.97	0.78	0.34	5.84	4.7	15.8	
C14:0 2-OH									
C15:0	0.34	0−1.7	3.51	0.80	1.12	1.03	3.2	1.56	
C15:1*ω*8 *c*								0.22	
C15:1*ω*6 *c*				0.12					
C16:1*ω*11 *c*								5.46	
**C16:1*ω*7 *c***	**40.97**	**10.6−23.1**		**13.83**	**18.13**	**10.33**	**54.2**	**47.3**	**26.9**
C16:1*ω*6 *c*		3.9−12.3			8.67			8.03	8.7
C16:1*ω*5 *c*		3.2−9.0		0.37	5.95		4.2		28.3
C16:1*ω*5 t		1.8−6.0			0.19				
C16:1*ω*9 t					3.91				
**C16:0**	**47.75**	**33.5−56.0**	**63.67**	**54,38**	**17.73**	**62.05**	**23.7**	**19.6**	**30.9**
C16:1*ω*9 *c*					33.01	7.36	6.5		
C16:1*ω*10 *c*									2.4
C16:1			11.90		0.80				
iC16:0 3-OH						3.96			
C16:0 3-OH	1.64		0.64	0.28		2.93	2.6	1.78	
9-ο-Me-C16:0		0−14.0	4.62						
C17:0 *cyc*	5.90		8.99	26.33					
C17:0			0.68	0.46	0.26				
C17:1			0.34						
C17:1*ω*6 *c*		0−1.8	0.43	0.62					
C17:1*ω*7 *c*			0.26						
C17:1*ω*8 *c*					0–16				
9-o-Me-C17:0			0.60						
11-0-Me-17:0		0−2.1	0.60						
C18:0	0.64		0.26		0.53				
C18:1		0−6.5	0.17		0.11				
C18:1*ω*7 *c*	1.49	0−2.9		0.71	0.93				1.7
C18:1*ω*9 *c*		0.6−1.8		0.62					
C19:0 *cyc*									
C19:1 *cyc*			1.37						
C20:0						2.66			
**Growth temp.** (°C)	**55**	**40**	**40**	**55**	**25**	**30**	**30**	**30**	**30**

Type Ib methanotrophs (moderately thermophiles, thermotolerants and mesophiles). Strains: **1**, Strain LS7-MC (the current study); **2**, *Methylococcus capsulatus* [41]; **3**, *Methylocaldum* sp. O-12 [17]; **4**, *Methylococcace* strain BFH1 [26]; **5**, *Methylotetracoccus oryzae* C50 C1 ^T^ [25]; **6**, *Methylogaea oryzae* E10 ^T^ [21]; **7**, *Methyloparacoccus murrellii* R-49797 ^T^ [22]; **8**, *Methylomagnum ishizawai* RS11 D-Pr^T^ [23]; **9**, *Methyloterricola oryzae* 73 a^T^ [24]. Values are given as a percentage of total fatty acids. Bold values show significant fatty acids for correlating among Type Ib methane oxidizers.

## Supplementary Materials

The following are available online at https://www.mdpi.com/2076-2607/8/2/250/s1. Table S1: Primers for amplification of functional genes of the strain LS7-MC. Table S2: Results of Southern blot analysis of radioactively labeled *pmoA* and *mmoX* probes. Table S3: Pairwise sequence (EMBOSS) alignment analysis of 16S rRNA gene sequences, *pmoA* gene and partial-derived PmoA amino acid sequences shows similarity between strain LS7-MC and other cultivated gammaproteobacterial methanotrophs. Table S4: Pairwise MxaF protein sequences similarity comparisons (EMBOSS) between LS7-MC and other related methanotrophs of the family *Methylococcaceae*. Figure S1: Phylogenetic tree based on 16S rRNA gene sequences. Figure S2: Phylogenetic tree based on 16S rRNA gene sequences. Figure S3: Phylogenetic tree of PmoA protein sequences. Figure S4: Phylogenetic tree of PmoA protein sequences.
