# Supplementary material for: A Novel Moderately Thermophilic Type Ib Methanotroph Isolated from an Alkaline Thermal Spring in the Ethiopian Rift Valley
Source: Microorganisms. 2020 Feb 13;8(2):250. doi: 10.3390/microorganisms8020250 (PMC7074724; doi:10.3390/microorganisms8020250)
Supplement: Supplementary file 1 [file microorganisms-08-00250-s001.pdf]

microorganisms

Table S1. Primers for amplification of functional genes of the strain LS7-MC.

Genes	Primer sequences (5′→3′)	Product(bp)	Results	Annealing temp. (°C)	Ref.
pmoA	A189f: 5'-GGNGACTGGGACTTCTGG-3'<br>Mb661: 5'-CCGGMGCAACGTCYTACC-3'	510	+	55	1
pmoA	*Pmo-F: 5'-GGGGACTGGGACTTSTGG-3'<br>*Pmo-R: 5'-GGAGCAACGTCTTTTACCGAARG-3'	508	+	55	6
mxaF	f1003: 5'- GCGGCACCAACTGGGGCTGGT-3'<br>f1561: 5'- GGGCAGCATGAAGGGCTCCC-3'	558	+	58	2
nifH	NifHf: 5'-GGHAARGGHGGHATHGGNAARTC-3'<br>NifHr: 5'-GGCATNGCRAANCCVCCRCANAC-3'	389	+	55	3
cbbL	McBCBBL 195F: 5'-CTGCTGACCGACCTCGACTA-3'<br>McBCBBL 706R: 5'-GTCACGTTGAGGTAGTGGCC-3'	500	+	58	4
	*f92: 5'-GGCTGCAGAGCTTYAMCTGG-3'<br>*Ar1430: 5'-CGCCTCCCTCRTACTGYTCGAG-3'	1335	-	55	6

*Probes used for Southern hybridization.

Table S2. Results of Southern blot analysis of radioactively labeled *pmoA* and *mmoX* probes.

Strain	*pmoA*	*mmoX*	Ref.
Methylococcaceae strain LS7-MC	+	−	This study
Methylococcus capsulatus Bath	+	+	7
Methylococcaceae strain BFH1	+	−	8
Methylacidiphilum kamchatkense Kam1	−	−	6

Table S3. Pairwise sequence alignment analysis of 16S rRNA gene sequences, *pmoA* gene and partial derived PmoA amino acid sequences shows similarity between strain LS7-MC and other cultivated gammaproteobacterial methanotrophs. Identity of PmoA sequences shows in the parentheses [9]. Values are given as a percentage. nr, not reported.

Strains name	16S rRNA	*pmoA*	PmoA
***Methylococcaceae* strain LS7-MC**	100	–	–
***Methylococcaceae* strain LS7-MC**	–	100	100
Methylococcus capsulatus Bath	92.7	87.8	99.4 (97.6)
Methylococcaceae strain BFH2	92.4	82.3	92.8 (87.4)
Methylocaldum szegediense OR2^T	92.0	79.7	92.3 (85.8)
Methylococcaceae strain BFH1	91.7	82.0	94.3 (88.0)
Methylococcaceae strain BRS-K6	91.6	84.0	93.2 (88.9)
Methylocaldum marinum S8^T	91.3	81.1	94.7 (88.8)
Methylococcaceae strain AK-K6	91.2	85.3	94.5 (89.6)
Methylocaldum gracile VKM 14L^T	90.9	82.7	94.7 (88.2)
Methylomagnum ishizawai RS11D-Pr^T	90.7	81.5	93.2 (88.2)
Methylogaea oryzae E10^T	90.7	79.0	95.3 (89.9)
Methyloterricola oryzae 73a^T	90.7	78.6	94.0 (87.4)
Methylocaldum tepidum LK6^T	90.6	80.2	92.9 (86.4)
Methylococcus thermophilus ACM 3585^T	90.5	nr.	nr.
Candidatus Methylospira palustris	90.5	80.3	93.4 (84.4)
Methyloparacoccus murrellii R-49797^T	90.3	84.6	96.2 (91.2)
Methylococcaceae strain GFS-K6	89.0	82.8	94.7 (88.1)
Methylohalobius crimeensis 10Ki^T	88.6	80.0	92.0 (82.8)
'Candidatus Methylloumidiphilus alinensis'	87.2	81.9	89.8 (85.6)
'Methylothermus' strain HB	87.0	74.6	92.2 (85.0)
Methylothermus thermailis MYTH^T	87.0	78.8	91.5 (85.6)
Methylothermus subterraneus HTM55^T	87.0	78.3	92.3 (86.4)
Methylovulum miyakonense HT12^T	85.6	71.0	92.9 (81.1)

Table S4. Pairwise MxaF protein sequences similarity comparisons between BFH1 and other related methanotrophs of the family *Methylococcaceae*. Identity of MxaF protein sequences shows in the parentheses [9]. Values are given in percentage.

Strains with accessions no.	*mxaF*	MxaF
**KP843192, *Methylococcaceae* strain LS7-MC**	100	100
AE017282, *Methylococcus capsulatus* strain Bath	85.7	97.8 (94.0)
KT921322, *Methylococcaceae* strain BFH2	85.6	99.3 (97.2)
GQ130269, *Methylococcaceae* strain BFH1	84.7	98.9 (95.4)
KP870209, *Methylococcaceae* strain AK-K6	84.5	97.2 (92.1)
HF954364, *Methyloparacoccus murrellii* R-49797^T	84.4	98.3 (91.7)
DQ002935, *Methylocaldum szegediense* strain O-12	84.0	96.9 (93.8)
KP870207, *Methylococcaceae* strain BRS-K6	83.7	97.6 (92.1)
DQ002936, *Methylocaldum szegediense* strain H-11	83.3	96.7 (93.4)
AJ868416, *Methylocaldum* sp. E10a	83.0	98.3 (92.8)
AJ868415, *Methylocaldum* sp. 5FB	82.8	96.6 (93.9)
KP870208, *Methylococcaceae* strain GFS-K6	82.3	96.4 (90.9)
AB453967, *Methylomarinum vadi* IT-4^T	82.3	95.7 (88.0)
AB501290, *Methylovulum miyakonense* HT12^T	80.0	94.0 (88.6)

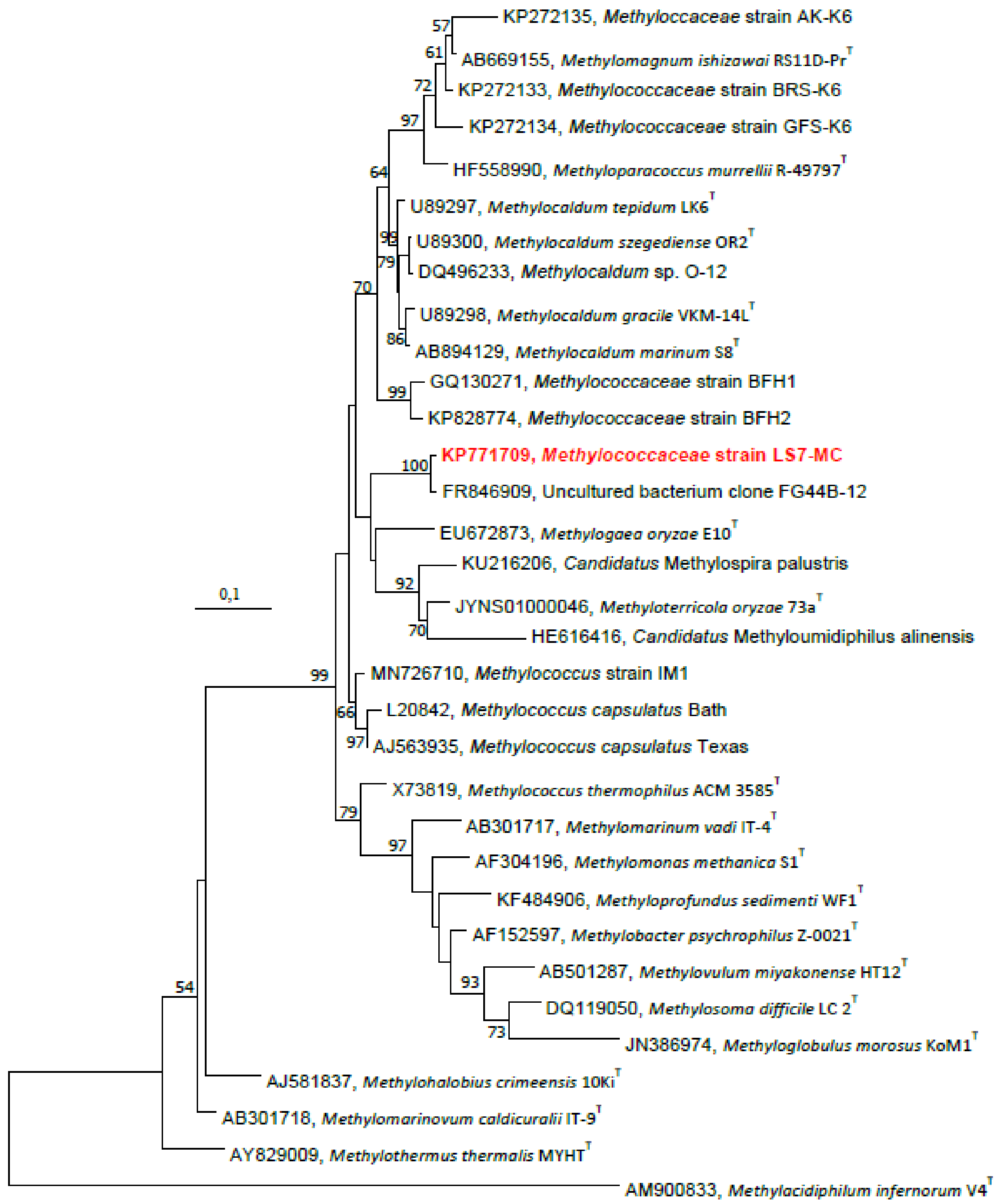

Figure S1. Phylogenetic tree based on 16S rRNA gene sequences. The tree is showing the relationship between the novel strain LS7-MC and other related gammaproteobacterial methanotrophs. The tree was inferred by a Maximum-Likelihood method based on the Kimura 2-parameter model. Evolutionary analyses were conducted in MEGA7 software package [10]. *Methylacidiphilum infernorum* V4 (AM900833) was used as an outgroup. Bootstrap values >50% are shown at nodes. The scale bar represents 0.1 changes per nucleotide position. GenBank accession numbers are given in front of the respective strains name.

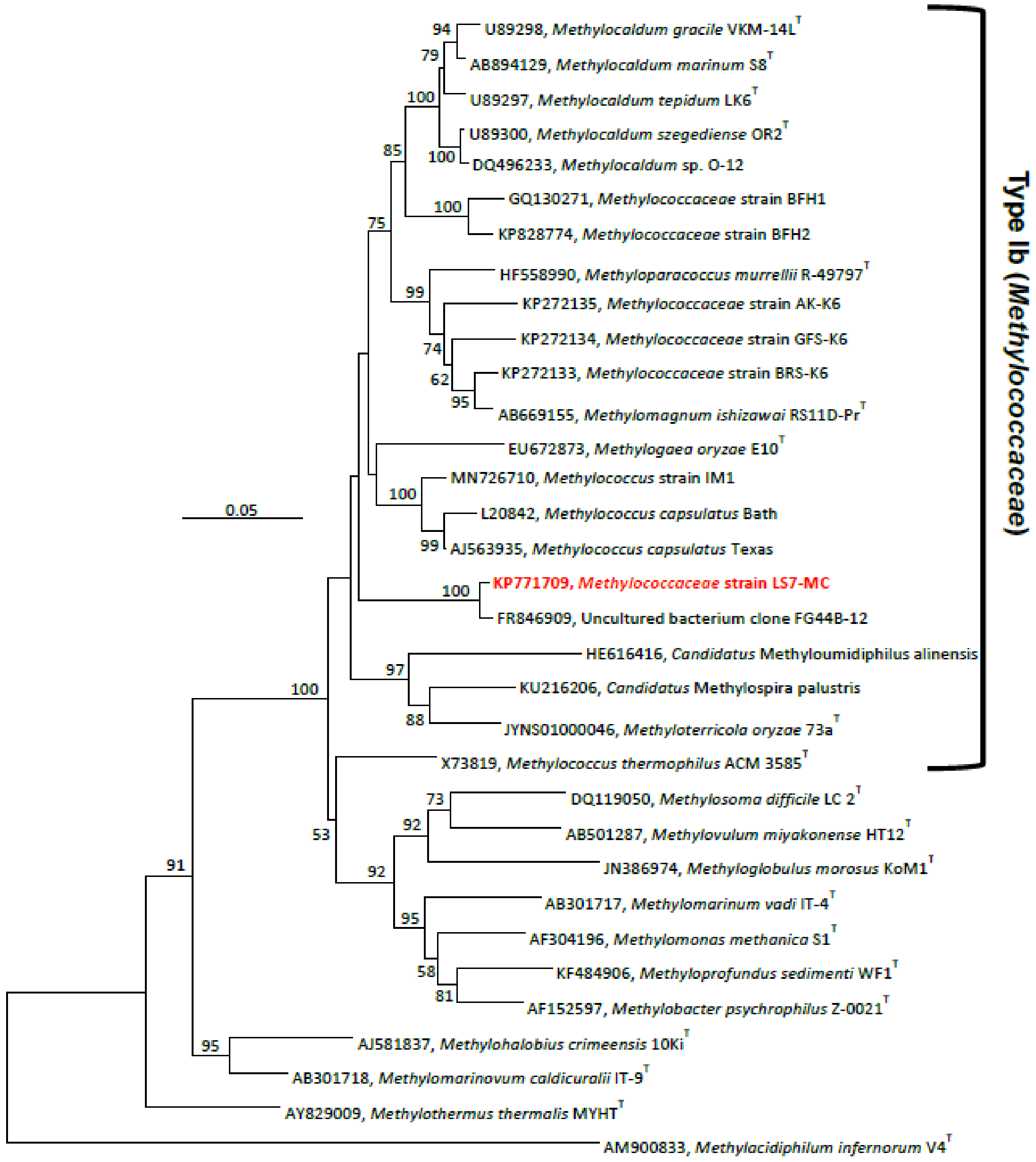

Figure S2. Phylogenetic tree based on 16S rRNA gene sequences. The tree is showing the relationship between the novel strain LS7-MC and other related aerobic gammaproteobacterial methanotrophs. The tree was inferred by a Minimum-Evolution method (MEGA7 software package). The evolutionary distances were computed using the Maximum Composite Likelihood model and are in the units of the number of base substitutions per site. The Neighbor-joining algorithm was used to generate the initial tree. Bootstrap values were determined using 1,000 replicates. *Methylacidiphilum infernorum* V4 (AM900833) was used as an outgroup. Bootstrap values below 50% are not shown. The scale bar represents 0.05 changes per nucleotide position [10]. .

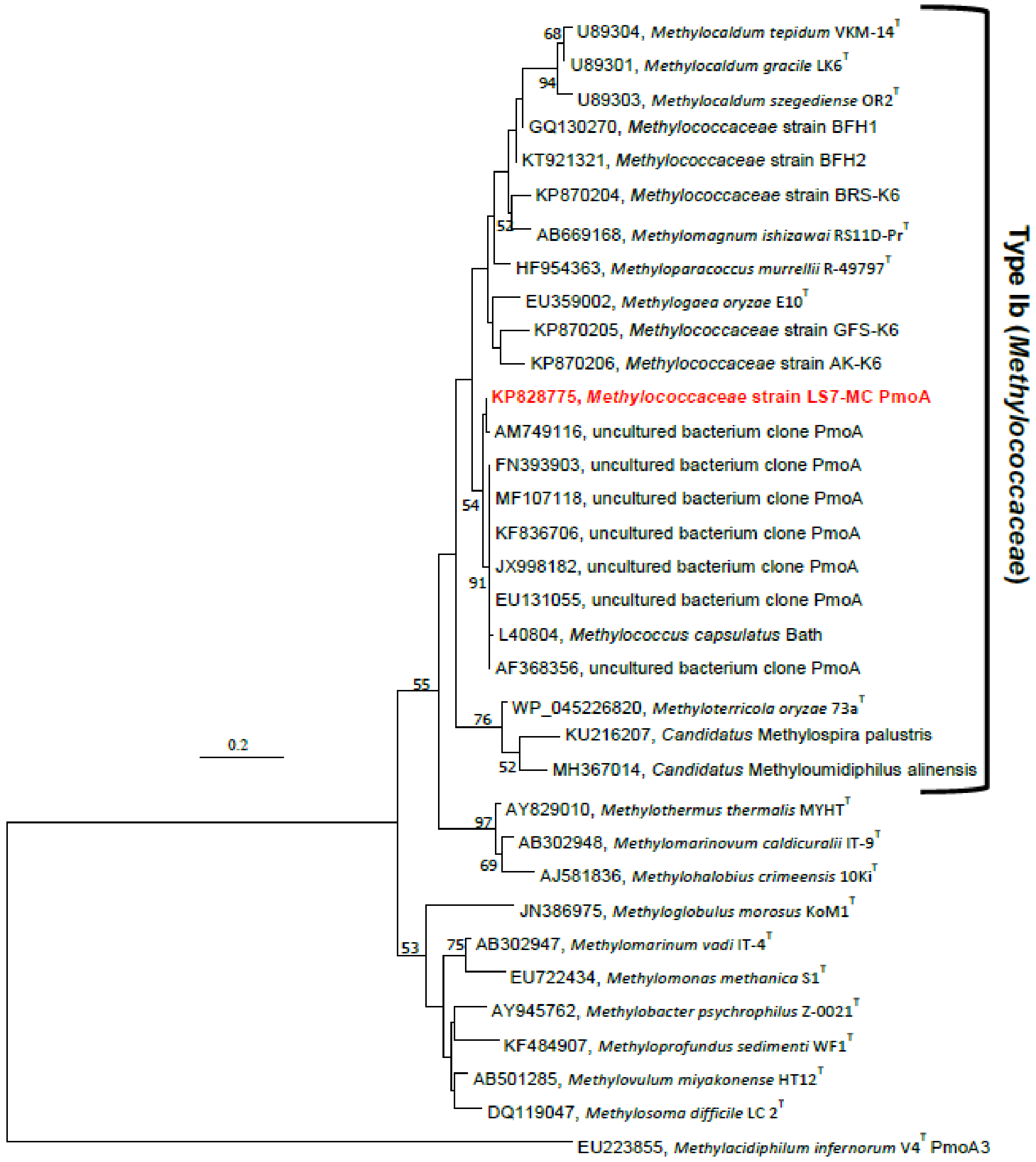

Figure S3. Phylogenetic tree of PmoA protein sequences. The tree is showing the relationship between the strain LS7-MC and other gammaproteobacterial methanotrophs. The evolutionary history was inferred by using the Maximum Likelihood method (deduced PmoA amino acid sequences) based on the JTT matrix-based model showing the position of strain LS7-MC and other described gammaproteobacterial methanotrophs. The tree was constructed using MEGA7 software package and was based on 131 amino acids. Bootstrap values were determined using 1,000 replicates. *Methylacidiphilum infernorum* V4^{T} PmoA3 (EU223855) was used as an outgroup. Bootstrap values below 50% are not shown [10]. .

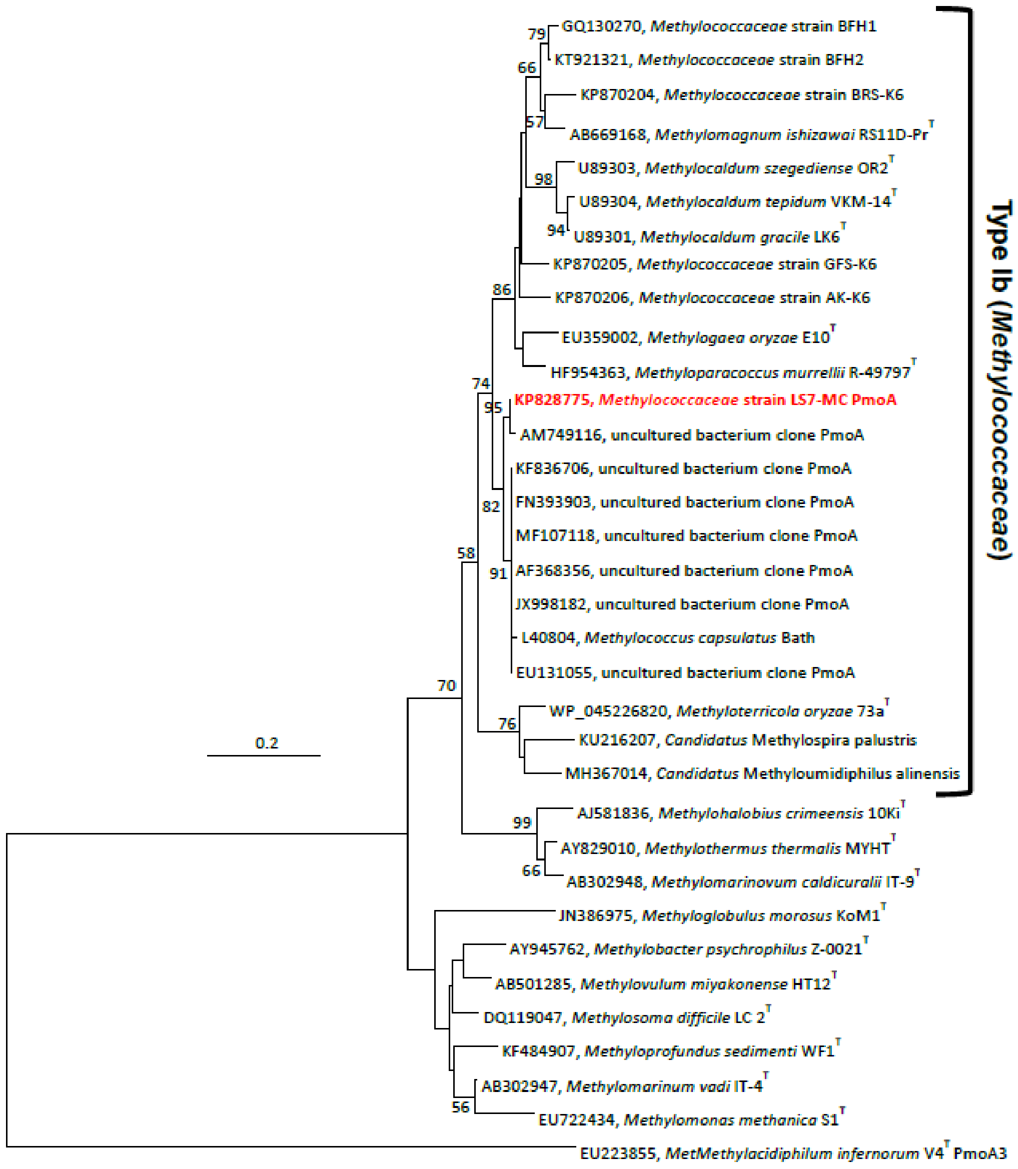

Figure S4. Phylogenetic tree of PmoA protein sequences. The tree is showing the relationship between strain LS7-MC and other related aerobic gammaproteobacterial methanotrophs. Minimum-Evolution tree based on deduced PmoA amino acid sequences showing the position of strain LS7-MC (with accession number) and other described gammaproteobacterial methanotrophic isolates. The tree was constructed using MEGA7 software package. The evolutionary distances were computed using the JTT matrix-based method and are in the units of the number of amino acid substitutions per site. Bootstrap values were determined using 1,000 replicates. *Methylacidiphilum infernorum* V4^{T} PmoA3 (EU223855) was included in the tree as an outgroup [10].

References:

1. Costello, A. M.; Lidstrom, M. E. Molecular characterization of functional and phylogenetic genes from natural populations of methanotrophs in lake sediments. *Appl. Environ. Microbiol*. **1999,** *65*, 5066−5074.
2. McDonald, I. R.; Murrell, J. C. The methanol dehydrogenase structural gene mxaF and its use as a functional gene probe for methanotrophs and methylotrophs. *Appl. Environ. Microbiol*. **1997**, *63*, 3218−3224.
3. Mehta, M. P.; Butterfield, D. A.; Baross, J. A. Phylogenetic diversity of nitrogenase (*nifH*) genes in Deep-Sea and Hydrothermal Vent Environments of the Juan de Fuca Ridge. *Appl. Environ. Microbiol*. **2003**, *69*, 960−970.
4. Baxter, N. J.; Hirt, R. P.; Bodrossy, L.; Kovacs, K., L.; Embley, T. M.; Prosser, J. I.; Murrell, J. C. The ribulose-1,5-bisphosphate carboxylase/oxygenase gene cluster of *Methylococcus capsulatus* (Bath). *Arch. Microbiol*. **2002**, *177*, 279−289.
5. McDonald, I. R.; Bodrossy, L.; Chen, Y.; Murrell, J. C. Molecular ecology techniques for the study of aerobic methanotrophs. *Appl. Environ. Microbiol*. **2008**, *74*, 1305−131.
6. Islam, T.; Jensen, S.; Reigstad, L. J.; Larsen, O.; Birkeland, N. K. Methane oxidation at 55 degrees C and pH 2 by a thermoacidophilic bacterium belonging to the *Verrucomicrobia* phylum. *Proc. Natl. Acad. Sci. USA* **2008**, *105*, 300–304.
7. Bodrossy, L.; Kovacs, K. L.; McDonald, I. R., Murrell, J. C. A novel thermophilic methane-oxidising γ-*Proteobacterium*. *FEMS Microbiol. Lett*. **1999**, *170*(2): p. 335–341.
8. Islam, T.; Torsvik, V.; Larsen, Ø.; Bodrossy, L.; Øvreås, L.; Birkeland, N. K. Acid-tolerant moderately thermophilic methanotrophs of the class Gammaproteobacteria isolated from tropical topsoil with methane seeps. *Front. Microbiol.* **2016**, *7*, 851.
9. The European Molecular Biology Open Softwear Suite. Emboss Tools For Sequence Analysis. Available online: **http://www.ebi.ac.uk/Tools/emboss/** (accessed on 26 August 2019).
10. Kumar, S.; Stecher, G.; Tamura, K. MEGA7: Molecular evolutionary genetics analysis version 7.0. *Mol. Biol. Evol.* **2016**, *33*(7), 1870–1874.
